# The case control studies of HIV and Intestinal parasitic infections rate in active pulmonary tuberculosis patients in Woldia General Hospital and Health Center in North Wollo, Amhara Region, Ethiopia

**Published:** 2015-06-10

**Authors:** Ambachew Woreta Hailu, Solomon G/Selassie, Yared Merid, Addis Adera Gebru, Yonas Yimam Ayene, Markos Kidane Asefa

**Affiliations:** 1 Woldia University, P.O.Box 400 Woldia, North Wollo, Amhara Region, Woldia, Ethiopia; 2 Addis Ababa University, School of Medicine, Department of Microbiology, Immunology and Parasitology; 3 Hawassa University, College of Medicine, Medical Microbiology Unit, Hawassa University, Department of Microbiology, Hawassa, Ethiopia; 4 Woldia University, Faculty of Health Sciences, Department of Nursing, P.O.Box 400, Woldia, North Wollo, Amhara Region, Ethiopia; 5 Woldia University, Faculty of Health Science, Department of Nursing, P.O.Box -400, Woldia, North Wollo, Amhara Region, Woldia, Ethiopia; 6 Huwawei company, Addis Ababa Woreda Net project, Addis Ababa, Ethiopia

**Keywords:** Association, Case-control, Co infection, TB/HIV

## Abstract

Tuberculosis remains a major health problem worldwide in the era of HIV/AIDS. Co-infection with intestinal parasites has been suggested to worsen the outcome of infection in addition to HIV infection. Hence, adequate information on TB patients with HIV and intestinal parasites infection is being needed to tackle the problem, undertake the integrated prevention and control program. This study was aimed to assess the prevalence of HIV and intestinal parasitic infections in active pulmonary tuberculosis patients compared with their healthy extended family of the subject as a control. A case-control study was carried out from November, 2010 to June, 2011 in Woldia General Hospital and Woldia Health Center. Stool sample were examined using direct technique and formol-ether concentration techniques. Modified acid fast stain was used to identify Oocysts of Cryptosporidium species and Isospora belli. HIV rapid tests were used to screen sero prevalence and AFB smear microscopy for screening Pulmonary TB patients. A total of 100 smear positive TB patients and 168 familial contacts were participated and the overall prevalence of intestinal parasite among TB patients was 49%; compared to 23.2% of the control. 41% of TB patients and 23.8% controls were found to be HIV infected. Double infection with both intestinal parasite and HIV was found in 61% TB patients and 52.5% of the controls. The proportions of TB patients infected with 1, 2, or more species of worms were 73.5%, 26.5% respectively; 82%, 18% were in controls and the odds of being an active TB patient is increased with the number of species of intestinal parasites the person harbors. The current study showed that a significant association between TB/HIV infection and intestinal parasite particularly Strongyloides stercoralis Cryptosporidium parvum and Isospora belli.

## 1. INTRODUCTION

Tuberculosis (TB) is the most common serious opportunistic infection in HIV positive patients and is the manifestation of AIDS in more than 50% of cases in developing countries [1,2]. Furthermore, TB shortens the survival of patients affected with HIV infection, accelerate the progression of HIV and is the cause of death in one third of people with AIDS worldwide [3,4,5]. TB is a leading cause of illness and death among people living with HIV in resource-poor and endemic areas of the world [6]. In 2008, there were an estimated 8.9–9.9 million incidence and 9.6–13.3 million prevalence of TB, 1.1–1.7 million deaths from TB among HIV negative people and an additional 0.45–0.62 million TB deaths among HIV-positive people. Among the incident cases of TB 30% cases is in Africa [7]. It is estimated that there were 9.4 million new TB cases in 2008 of which 1.4 million cases were recorded among people living with HIV. In the same year the year 1.8 million people died due to tuberculosis all over the world, including 500,000 with HIV co-infection [8]. Pulmonary tuberculosis in African HIV-positive patients presents with a spectrum of radiographic abnormalities predictive of stage of HIV disease progression (9). It is estimated that over 40 rapid serologic TB tests that use various antigenic compositions to detect patients’ antibodies are currently commercially available in many low- and middle-income countries. These may be suitable to diagnose TB in primary health-care settings with a test result is available in less than 15 minutes [10]. In addition, Antigen-antibody reactions are visualized on the lines using anti-human antibody bound to substances such as colloidal gold. The test takes minutes to perform. [10].

Parasitic infections affect the nutritional status of the individual leading to immunological alterations that promote a decrease in the efficacy of the immune response, favoring the occurrence of other bacterial infections [11]. Tuberculosis and enteroparasitosis affect primarily low social and economic level populations, living clustered in precarious habitational settings [12]. Progress in implementation of interventions to reduce the burden of TB in HIV-positive people has continued; in 2008, 22% of TB patients knew their HIV status (up from 20% in 2007) including 45% of patients in the African Region. One of the interesting aspects of interaction between intestinal parasitic infection and tuberculosis is the parasitism influence in cellular response to *M. tuberculosis* [11]. Some *in vitro* studies suggested that parasitized individuals had less effective cellular response compared to non parasite. Therefore, intestinal helminthes infection may be one of the risk factors for the development of active pulmonary TB in addition to HIV infection [11].

In Ethiopia, there are a few reports about the interaction of HIV and parasite in TB patients [13, 14, 15, 16]. However their work is limited to few samples and site. As a result, adequate information on TB patients with HIV and intestinal parasites in such cases is in scarce. Hence, adequate information on TB patients with HIV and intestinal parasites infection is being needed to tackle the problem, undertake the integrated prevention and control program. Ethiopia is among the 27 high MDR-TB burden countries with 1.6% (0.9–2.7) among newly registered TB cases with MDR-TB and 11.8 % (6.4–21.0) MDR among previously treated TB cases (95% CI) (WHO, 2010). Multidrug-resistant TB (MDR-TB) is a form of TB that is difficult and expensive to treat and fails to respond to standard first-line drugs. Extensively drug-resistant TB (XDR-TB) occurs when resistance to second-line drugs develops on top of MDR-TB (17). All patients tested negative for HIV infection. Of the 211 patients, 21 patients (10%) showed resistance to one of the fluoroquinolones and 14 (6.6%) showed resistance to the injectable agent, kanamycin. Five cases of XDR-TB were detected. Thus, the prevalence of XDR-TB among MDR-TB patients was 2.4 per cent. Among the five XDR-TB patients, two patients had a definite history of previous treatment with second-line drugs; the remaining three patients had multiple previous courses of anti-tuberculosis therapy which most likely included second-line agents [18]. The application of Directly-Observed Treatment, Short-course (DOTS), the universally accepted intervention for TB treatment, is crucial in AIDS cases. However, the DOTS strategy includes not only the observation of the patient’s medicine intake but also other important issues that constitute a strategy launched in 1996 by the WHO [19, 20]. Whole blood and/or bone marrow specimens are collected only if disseminated TB is suspected, mainly in patients with an underlying severe immunosuppressive condition such as AIDS. Bone biopsies are the specimen of choice when skeletal TB is suspected. In general, AFB smear microscopy from body fluids is rarely positive and the whole sediment from concentrated specimens should rather be cultured [21). Specimens should be collected in sterile, leak-proof containers and labeled with the patient’s name and/or identification number before anti-tuberculosis chemotherapy is started. Induced sputum specimens should be labeled as such because they resemble saliva and may be disregarded at the laboratory [21]. Smear staining is based on the high lipid content of the cell wall of Mycobacteria which makes them resistant to decolourization by acid-alcohol after the primary staining. To determine that a clinical specimen contains AFB, the specimen is spread onto a microscope slide, heat-fixed, stained with a primary staining, decolorized with acid-alcohol solution and counter stained with a contrasting dye in order to obtain better differentiation between the microorganism and the background. The slide is observed under the microscope for the detection of AFB [22].

In 2008, an estimated 1.9 million people living in sub-Saharan Africa became newly infected with HIV, bringing the total number of people living with HIV to 22.4 million. While the rate of new HIV infections in sub-Saharan Africa has slowly declined—with the number of new infections in 2008 approximately 25% lower than at the epidemic’s peak in the region in 1995—the number of people living with HIV in sub-Saharan Africa slightly increased in 2008, in part due to increased longevity stemming from improved access to HIV treatment. Adult (15–49 year old) HIV prevalence declined from 5.8% in 2001 to 5.2% in 2008 [23]. [15] found the 71% prevalence of intestinal helminthes particularly, *A. lumbricoides*, hookworm and *S. stercoralis* were independently and significantly associated with active TB and suggested that of intestinal helminthes infection may be one of the risk factors for the development of active pulmonary TB in addition to HIV infection. Other related investigation in a country by [14] showed prevalence of intestinal parasites in the TB patients was 40.5%. The parasites were detected in 37.3% of HIV sero positive and 43.9% of HIV-sero negative TB patients. The main Aim of this study was to assess the prevalence of HIV and Intestinal Parasitic Infections in active pulmonary tuberculosis patients and associated risk factor in Woldia General Hospital and Woldia Health Center, Woldia, North Wollo, Amhara Region, Ethiopia.

## 2. MATERIALS AND METHODS

Woldia Town is the capital of North Wollo, Amhara Region, Northeast Ethiopia with latitude and longitude of location of 11° 50’N 39036’0”E and an elevation of 2112 meters above sea level. It is located 520 Km away from Addis Abeba. Woldia General Hospital (WGH) and Woldia Health Center (WHC) are the health institutions involved in the study. Two hundred (74.6%) and 68 (25.4%) of the study subjects were from WGH and WHC respectively (Fig 1). The study was conducted from November 2010 up to June 2011. A case control study was conducted to assess the prevalence of HIV and intestinal parasite infections in active pulmonary tuberculosis patients in Woldia General Hospital and Woldia Health Center in North Wollo Amhara Region, Ethiopia. The study population was consisting of active pulmonary TB patients, who were attending TB clinic in WGH and WHC. The report from WGH and WHC, number of patients in treatment, newly registered pulmonary cases were very few during the study period. Therefore, consecutive clients were included in the present study within the study period, from November 2010 to June 2011 in Woldia General Hospital and Woldia Health center. An adult (age >18 years) newly diagnosed pulmonary TB (smear positive) and healthy household contacts were included. New TB patients are those patients who did not take anti-TB drugs before or have taken it for less than 30 days were included. Patients received anti-parasitic treatment and study subjects who were unwilling to give consent were excluded from the study. All eligible consecutive patients who visited the TB clinic were included in the study within the study period. The result of AFB smear microscopy and HIV testing from routine existing health service system in the country were utilized for the study. Additionally, Stool samples were taken from pulmonary TB patients. The household (in this study, household was defined as the extended family living together in the same area and eating from the same pot) of each case was visited and information collected on various demographic and socioeconomic variables.

## 2.1 Direct microscopy

A fresh faecal specimen, uncontaminated with urine and collected into a suitable size, clean, dry, leak-proof container were collected. A large teaspoon amount of faeces or about 10 ml of a fluid specimen were collected using sterile container but being free of all traces of antiseptics and disinfectants (also a bedpan if this is first used to collect a specimen from an inpatient). Three stools were specimens collected on 3 consecutive days and parasitological examined on the same day by direct microscopy and formol-ether concentration [24].

## 2.2 Formol-ether concentration technique

The direct examination of faeces is essential to detect motile parasites and is usually adequate to detect significant helminth infections. However, when the number of ova of parasite very few to detect a concentration technique should be performed [24].

## 2.3 Modified acid-fast method

**Oocysts** of Cryptosporidium and Isospora belli can be detected in wet preparations but they are more easily identified in smears stained by the modified Ziehl-Neelsen (Zn) method following concentration by the formol ether Oocysts concentration technique as described in the following diagram [24].

## 2.4 Quality control and Data analysis

Reagents were checked by known positive and negative samples from the clinic before stool sample preparation and examination. All specimens were also checked for their label and quality. Data were analyzed using SPSS evaluation version 15. Different characteristics of study participants were described using mean, median, standard deviation, range and percentage. Statistical significance of differences in proportions was evaluated by Pearson's Chi-square test. P value <0.05 were considered significant.

The study was approved by the Institutional Ethics Review Board of Hawassa University College of Health Sciences. Informed written consent was obtained from each study subject. The result of AFB smear microscopy and HIV testing from routine existing health service system in the country were utilized for the study. Confidentiality was maintained throughout the study. Patients’ positive for parasites, HIV and TB were sent to the appropriate physicians for treatment and follow up.

## 3. RESULTS AND DISCUSSION

### 3.1 Description of Study Participants

A total of 100 smear positive TB patients and 168 familial contacts of the cases were asked to get involved in study. Of the TB patients, and control subjects, 45(45%) and 85 (50.6%) were females, respectively. The age of the subject was within the range of 18–65 years and the mean age of the respondents (PTB patients) was 37 years. Likewise, the mean age of the TB cases was similar to that of the controls. Concerning the educational status, 44% were without formal education, 44% were with basic education and 12% were with post basic education. Likewise, the educational status of the control group was 28%, 56%, 16% respectively. About 43 of study subjects were married, 20 were single, 21 were divorced, and 16 were widowed where as 50%, 24.4 %, 14.3 %, and 11.3 % of the control group with the same pattern of distribution. from the total study participants, 43%, 29% of them were farmers, 7%, 6.5% were students, 15%,11.9% were private workers (including daily laborers), 6%, 10.1% were merchants, 13%, 17.9% were governmental employee, 6%, 13.1% were house wives, 4%, 7.1% were prisoners and 6%, 3.6% were others types of occupation (like drivers, dependants, self employee, etc), of case and controls respectively.

### 3.2 Distribution of Parasitic infection versus Sociodemographic Variables of Study Participants

The overall prevalence of intestinal parasite among TB patients who were enrolled in the study was 49 % whereas the prevalence of the in the control group was 23.2 % (Table 1). The difference was statistically significant (P = 0.000). 41% TB patients and 40 (23.8%) controls were HIV infected. The difference was statistically significant (P < 0.003). Double infection with both intestinal parasite and HIV was found in 61% (25/41) of TB patients and 52.5 % (21/40) of the controls (OR, 1.414, 95% CI (0.585–3.417). The proportions of TB patients infected with 1, 2, or more species of worms were 73.5%, 26.5% respectively; in controls these proportions were 82%, 18% (Table 2). The prevalence of intestinal parasite in the different age groups of TB patients compared with controls. The mean (±SD) age of TB patients infected with intestinal parasite were 38.2 (±11.25) and the mean age of intstinal parasite positive controls were 36.33 (±12.34). Likewise, among TB patients with intestinal parasitic infection 49 % (24/49) of them were male while 51% (25/49) were female and this proportion in controls were; 51.3% (20/39), 48.7% (19/39) respectively. In general there were no significant difference in worm infection rate in relation to age and sex groups (table 3). The prevalence of worm infection in HIV-negative TB patient (40.7%) was significantly different with that of HIV-positive TB patients (61 %) (P = 0.048). In the present study Worm infection rates in HIV-positives (52.5 %) and HIV-negative controls (14.1%) were significantly different (P = 0.000).

### 3.3 Sociodemographic Variables versus TB status of Study Participants

Results from univariate analysis carried out on the 268 cases/household control are displayed in Table 5. As can be seen, gender was not found to increase the risk of TB in the Study (P=0.376) while age group was not linked to risk of TB. Being married, Widowed/divorced or living as a single individuals were not associated with the risk of developing TB in study. Even if the study subjects with no formal education more likely to increase the risk of TB than basic and post basic education, Although this effect was not statistically significant (P=0.27). Although the odds of being a farmer as a risk factor for TB increased than housewife member of the family, but this effect also showed no statistical significance (P=0.15).

*Cryptosporidium parvum, Isospora belli, Strongyloides stercoralis* showed statistically significant association with HIV status among TB patients, while *Entamoeba histolytica, Giardia lamblia, Ascaris lumbericoides, Trichuris trichuria, Entrobius vermicularis*, Hookworm were not associated with HIV among TB patients in study area (Table-5).

The current study showed that intestinal parasitic infections were more prevalent (49%) in active pulmonary TB patients than their healthy familial contacts (23.2 %). The overall prevalence reported in this study is lower than those studies conducted previously in other study areas. A study from Gondar Teaching Hospital, Northwest Ethiopia also indicated the association of intestinal parasite with Pulmonary TB patients with prevalence rate of 71% [15]. Other studies also reported a higher rate of intestinal nematodes (40.5%, 57.8%) in patients with pulmonary TB compared to controls [13]. These decreasing trends of intestinal parasitic infection rate may be associated to the possible ecological changes as well as number of sampling and site selection may be the possible reason.

The association between TB and worm infection observed in this study could be confounded by poor living conditions. Indeed, poor living condition and malnutrition are important predisposing factors to TB [15; 26] and it is possible that the poorer people were more likely to get infected with worms and also more likely to get TB. However, the fact that our controls were selected from the same households, living under the same socio-economic conditions as the patients, reduces the possibility that the observed association is confounded by poor living condition. The present study indicates association between worm infection and TB. Several studies also reported that helminthes infection could increase susceptibility to TB. In the present study the association between the intestinal parasite species and active TB were evaluated and the result showed that the odd of being an active TB patient is increased with the number of species of intestinal parasites the person harbored. This could highly support the hypothesis that intestinal parasitic infection may be an important risk factor for the development of active TB. This idea is consistent with this study of [15] The sero prevalence of HIV was 41% and 23.8% in smear positive TB patients and controls respectively in the current study (P < 0.003). The published reports about sero prevalence of HIV among tuberculosis patients are highly variable in Ethiopia. The WHO global report 2008 estimates that in Ethiopia 40% TB patients tested for HIV are HIV positive which was similar with current study. However, the prevalence rate reported from this study was slightly lower than a report of 52.1% HIV prevalence among TB patients from the northern part of Ethiopia [14] and higher than a previous report of 19% from the southern part of Ethiopia [14].

It is indeed a valid argument that the higher intestinal parasitic infection rate in TB patients could be due to HIV induced immunosuppression that could incline patients to getting different types of infections including intestinal parasite. Consistent with the argument, the finding from this study showed that a significant association between opportunistic parasite infection particularly *Strongyloides stercoralis* and *Cryptosporidium parvum* and TB/HIV co infected patients. To examine this possibility, In the present study Worm infection rates in HIV-positives (52.5 %) and HIV-negative controls (14.1%) were significantly different (P = 0.000), which showed that the association between *Giardia lamblia* and *Trichuris trichiura* with TB might be confounded by the high HIV infection rate in TB patients. On contrary, other studies reported that worm infection was associated with active TB independently of HIV infection [15]. In the current study, infection with both intestinal parasite and HIV was found in 61% (25/41) of TB patients. [13] was also reported the synergistic effect of HIV and intestinal parasite among TB patients with the prevalence rate of 37.3%. Further, the previous study by [13] indicated that IgE level was significantly higher in TB patients co infected with intestinal helminthes and HIV compared to those infected without co infection. The highly elevated IgE profile in TB patients with HIV co infection might be a result of HIV induced immune dysregulation which induces shift in cytokine production from Th1 to Th2 [25]. In this study, age and sex were not associated with having active TB. Another reports also showed that age and sex were not found to have association with smear positive TB patients [15, 16] which is consistent with the current data. However, some studies reported that Male sex and increasing age were significant risk factors for TB). The reason by which no association was not observed between sex of the subjects and having active TB was probably due to the similar number of male and females among the study population were diagnosed or a true background sex difference is difficult to assess or there may not be considerable sex differences with regard to stigma in the study area which may result in differential health seeking behavior and access to care between males and females. Marital, occupation and educational status were not found to increase the risk of TB in present study. [26] showed absence of association between education levels, occupation, unemployment and TB consistent with current data. This is contrary to the results of other studies. While other studies have shown that marital status affects the risk of TB, with single men having a greater risk of TB than married men and people living without children, alone or with adults of their own sex only, have higher risks of developing TB than people living in households with children However, the present study conducted where married/coupled study subjects outnumbered the single and widowed/divorced and this in turn the fact that married subjects have a stable life style that reduced their socioeconomic difficulties which is the major risk for TB.

## 4. CONCLUSION

This study revealed that the prevalence TB/HIV co infection of 41% amongst active TB patients that of 23.8% amongst the control group. Likewise, 49% of active TB patients were find to be co infected with different intestinal parasite compared to 23.2% co infection amongst the control. In this study, HIV infection was more prevalent among TB patients than controls. Some opportunistic parasitic infection such as *Cryptosporidium parvum, Isospora belli* and *Strongyloides strecoralis* were showed a significant association with HIV status among TB patients. Some socio-demographic risk factors such as age, sex, occupation, educational level and marital status were not found to be associated with TB. The higher prevalence of intestinal parasite among TB patients in the present study calls for an integrated plan treatment, preventions and control program targeting both parasite and TB in the study area. Exhaustive study on correlation between individual parasite with co infection. A further clinical and immunological study in an area with high prevalence of intestinal parasite infection is required to elucidate the impact of these parasites on the development of active TB. This study should be done on the synergetic effect of HIV and intestinal parasite on the pathogenesis of TB.

## Figures and Tables

**Figure 2:**
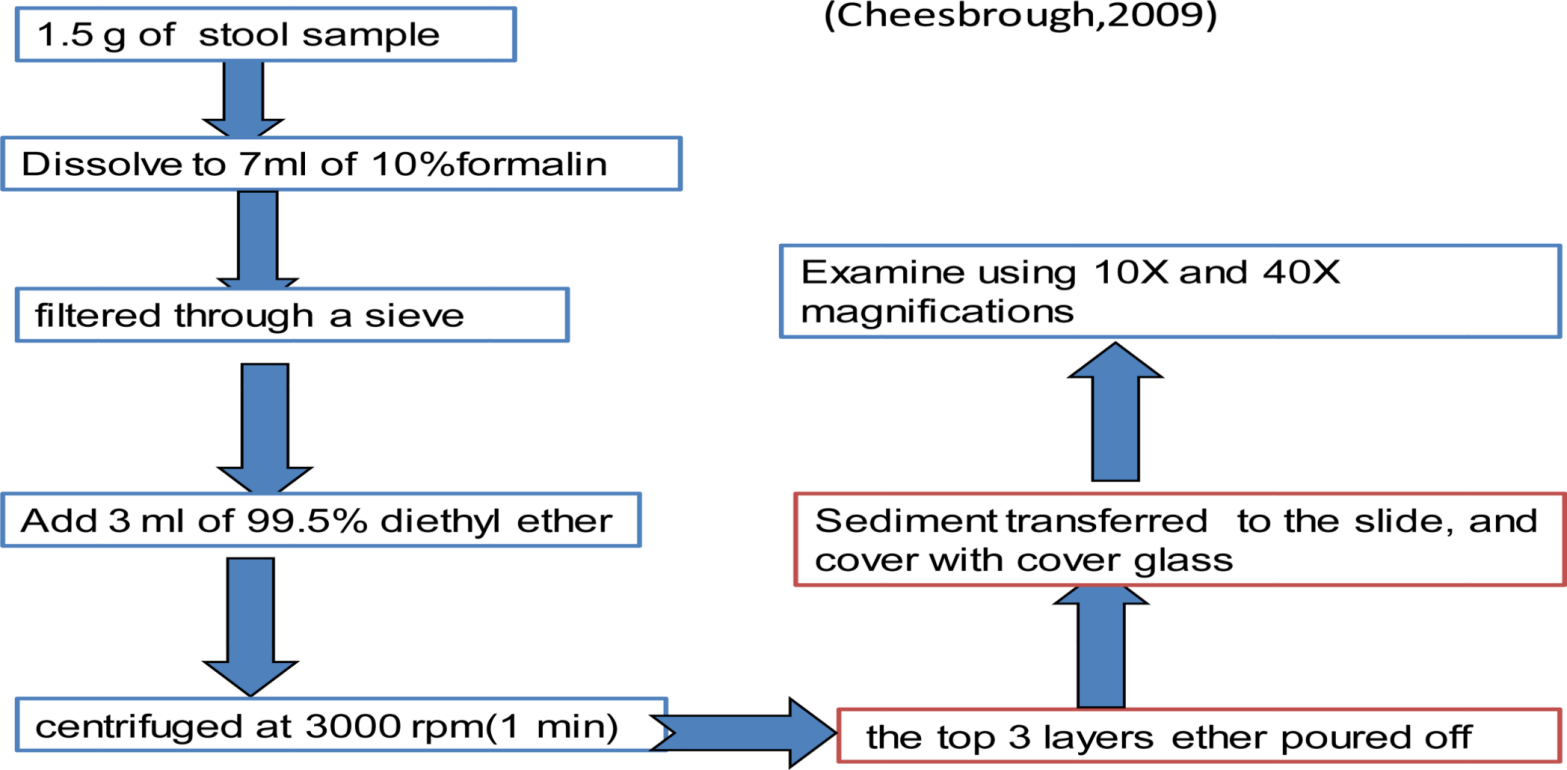
Formol-ether concentration from the collected sample

**Figure 3:**
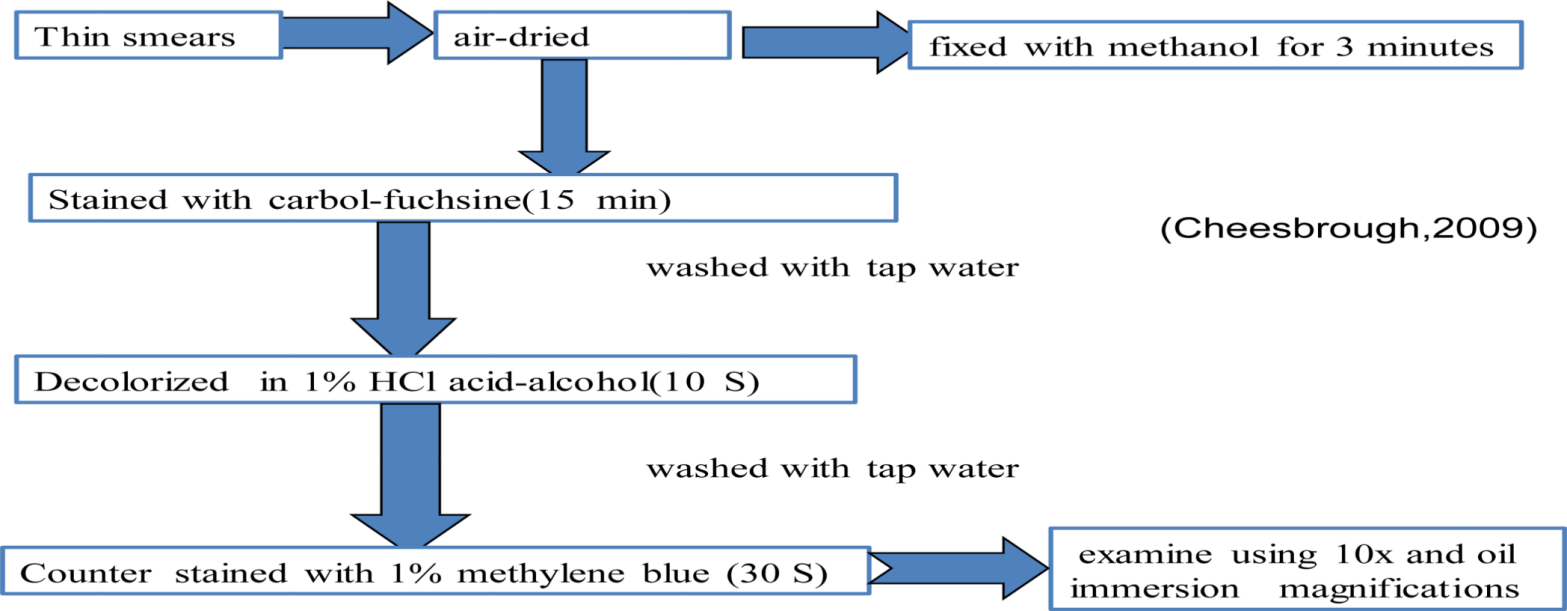
Modified Ziehl-Neelsen (Zn) method from the collected sample

**Table 1:** Single and double infection in TB patients and controls in Woldia general hospital and Woldia health in north Wollo, Ethiopia, November – June, 2011

Infection	TB patients № (%)	Controls № (%)	P- value
**Single infection**	36 (73.5)	32(82)	0.000
**Double infection**	13(26.5)	7(7(18)	0.008
***Entamoeba histolytica / Giardia lamblia***	4(31)	2(28.6)	
***Entamoeba histolytica/* Hookworm**	0	1(14.3)	
***Entamoeba histolytica / Cryptosporidium parvum***	1(7.7)	0	
***Entamoeba histolytica/ Strongyloides strecolaris***	0	1(14.3)	
***Entamoeba histolytica/ Entrovious vermicularis***	0	1(14.3)	
***Giardia lamblia / Cryptosporidium parvum***	2 (15)	0	
***Ascaris lumbericoides / Trichuris trichuria***	4(31)	1(14.3)	
***Ascaris lumbericoides/Giardia lamblia***	1(7.7)	0	
***Giardia lamblia/* Hook worm**	1(7.7)	0	
***Entamoeba histolytica/ Isospora belli***	0	1(14.3)	
**Over all**	49(100)	39(100)	

**Table 2:** The prevalence of intestinal parasite recorded from pulmonary TB and control specimen in Woldia General Hospital and Woldia Health Center in North Wollo, Ethiopia, and November – June, 2011

Parasite Detected	TB status		OR	P-Value
			
	Smear positive TB *n* (%)	Controls *n* (%)		
***Entamoeba histolytica***	9(9.0)	13(7.7)	1.18	0.72
***Giardia lamblia***	15(15.0)	12(7.1)	2.29	0.04
***Cryptosporidium parvum***	6(6.0)	3(1.8)	3.51	0.06
***Isospora belli***	3(3.0)	1(0.6)	5.17	0.12
***Ascaris lumbericoides***	8(8.0)	7(4.2)	2.0	0.19
***Trichuris trichuria***	13(13.0)	1(0.6)	24.95	0.000
***Strongloides strecolaris***	5(5.0)	3(1.8)	2.90	0.14
***Entrovious vermicularis***	2(2.0)	2(1.2)	1.69	0.60
**HIV1/2**	41(41)	40(23.8%)	2.22	0.003
**Any worm infection**	49(49)	39 (23.2%)	3.18	0.000

No: number of positive subjects; OR, odds ratio.

**Table 3:** Distribution of parasitic infection in different age group and in both sexes of TB patients and controls in Woldia General Hospital and Woldia Health Center in North Wollo, Ethiopia, and November – June, 2011

Age and sex of cases and controls	Case		Control	

	Infected	OR (95% CI)	Infected	OR (95%CI)
**Sex**				
**Male**	24(49.0)	1	20(51.3)	1
**Female**	25(51.0)	0.62(0.28–1.37)	19(48.7)	1.1(0.54–2.25)
**Age Group**				
**15–24**	7(14.3)	1	10(25.6)	1
**25–34**	12(24.5)	1.17(0.33–4.1)	7(17.9)	1.8(0.59–5.39)
**35–44**	13(26.5)	1.44(0.33–4.0)	10(25.6)	1.9(0.7–5.2)
**45–54**	14(28.6)	0.31(0.074–1.3)	9(23.1)	1.07(0.6–3)
**≥55**	3(6.1)	1.5(0.25–8.4)	3(7.7)	0.833(0.17–4.0)

OR= Odd Ratio

**Table 4:** Socioeconomic factor for TB: comparison of TB cases and household controls in Woldia general hospital and Woldia health in north Wollo, Ethiopia, November – June, 2011 (univariate analysis) (*n* = 268)

Socioeconomic factors	Case (n %)	Control (n %)	OR (95% CI)	bP-value
**Sex**				
**Male**	55	83(49.4%)	1	
**Female**	45	85(50.6%)	1.25(0.76–2.1)	P=0.376
**Age Group**				
**15–24**	15	34(20.2%)	1	
**25–34**	28	37(22%)	2.0(0.65–6.24)	P=0.597
**35–44**	30	56(33.3%)	1.2(0.40–3.42)	
**45–54**	19	32(19.1%)	1.7(0.58–4.74)	
**≥55**	8	9(5.4%)	1.5(0.49–4.54)	
**Occupation**				
**Farmer**	43	50(29.8%)	1	
**Merchant**	6	17(10.1%)	2.437(0.88–6.73)	P=0.153
**Governmental-employee**	13	30(17.9%)	1.985(0.92–4.28)	
**Private**				
**Housewife**	15	20(11.9%)	1.15(0.524–2.51)	
**Prisoner**	6	22(13.1%)	3.15(1.171–8.49)	
**Other**	4	12(7.2%)	2.58(0.775–8.59)	
**Student**	6	6(3.6%)	0.86(0.258–2.86)	
	7	11(6.6%)	1.351(0.48–3.79)	
**Education**				
**No formal education**	44	47(27.98%)	1	
**Basic education**	44	94(55.95%)	2.00(1.16–3.5)	P=0.27
**Post basic**	12	27(16.1%)	2.10(0.95–4.7)	
**Marital status**				
**Married**	43	84(50%)	1	P=0.375
**Single**	20	41(24.4%)	1.05(0.55–2.0)	
**Divorced/windowed**	37	43(25.6%)	0.61(0.34–1.0)	

OR, Odd Ratio

b *P*-value from Pearson Chi-Square test^x2^

**Table 5:** Type and Frequency of Intestinal helminthes in TB patients by HIV status status in Woldia general hospital and Woldia health in north Wollo, Ethiopia, November – June, 2011

Intestinal parasite species detected in stool	TB patients		
	
	HIV− *n* (%)	HIV+ *n* (%)	P-Value
*Entamoeba histolytica*	5(8.5)	4(9.8)	0.86
*Giardia lamblia*	8(13.6)	7(17.1)	0.628
Cryptosporidium parvum*	0 (0·0)	6(14.6)	0.002
Isospora belli*	0 (0·0)	3	0.035
*Ascaris lumbericoides*	4(6.8)	4(9.8)	0.59
*Trichuris trichiura*	10	3(7.3)	0.16
*Strongloides sterecoralis*	0 (0·0)	5(12.2)	0.006
Hookworm	0	1	0.23
*Entrobius vermicularis*	1	1	0.79

## References

[1] WHO (2007). Pathways to better diagnostics for tuberculosis: a blueprint for the development of TB diagnostics by the new diagnostics working group of the Stop TB Partnership, World Health Organization.

[2] Anthony MR, Rutitzky IL, Urban FJ, Stadecker JM, Gause CW (2007). Protective immune mechanisms in helminthes infection. National Review of Journal of Immunology.

[3] Correale J, Farez M (2007). Association between Parasite Infection and Immune Responses in Multiple Sclerosis. Neurology.

[4] Diane V, Havlir, Getahun H, Sanne I, Nunn P (2008). Opportunities and Challenges for HIV Care in Overlapping HIV and TB Epidemics. JAMA.

[5] Multidrug and extensively drug-resistant TB (M/XDR-TB): 2010 global report on surveillance and response.

[6] Wongstitwilairoong B, Srijan A, Piyaphong S, Khungvalert V, Chivaratanond O, Bodhidatta L (2005). Significantly increased recovery of intestinal parasites on routine stool specimen evaluation southeast. Asian Journal of Tropical Medicine Public Health.

[7] WHO (2009c). update Tuberculosis FACTS.

[8] WHO (2009d). Guidelines for HIV Diagnosis and Monitoring of Antiretroviral Therapy.

[9] itacco PL (2007). Tuberculosis. From basic science to patient care Tuberculosis.

[10] WHO (2008). Laboratory-based evaluation of 19 commercially available rapid diagnostic tests for tuberculosis, WHO (Diagnostics evaluation series, World Health Organization on behalf of the Special Programme for Research and Training in Tropical Diseases.

[11] Co TR, Hirsch CS, Toossi Z, Dietze R, Ribeiro-Rodrigues R (2006). Intestinal helminthes co-infection has a negative impact on both anti-Mycobacterium tuberculosis immunity and clinical response to tuberculosis therapy. British Society for Immunology, Clinical and Experimental Immunology.

[12] Karp CL, Auwaerter PG (2007). Co infection with HIV and tropical infectious diseases. II. Helminthic, fungal, bacterial, and viral pathogens. Clinical Infectious Diseases.

[13] Kassu A, Mengistu G, Ayele B, Diro E, Mekonnen F, Ketema D, Moges F, Mesfin T, Getachew A, Ergicho B, Elias D, Wondmikun Y, Aseffa A, Ota F (2007). HIV and intestinal parasites in adult TB patients in a teaching hospital in Northwest Ethiopia. Tropical Doctor.

[14] Kassu A, Mohammed A, Fujmaki Y, Moges F, Elias D, Mekonen F, Mengestu G, Yamato M, Wondemikun Y, Ota F (2004). Serum IgE levels of tuberculosis patients in a tropical setup with high prevalence of HIV and intestinal parasitoses. Clinical Experimental Immunology.

[15] Elias D, Mengistu G, Akuffo H, Britton S (2006). Are intestinal helminthes risk factors for developing active tuberculosis?. Tropical Medicine International Health.

[16] Elias D, Wolday D, Akuffo H, Petros B, Bronner U, Britton S, Hansen A (2001). Effect of deworming on human T cell responses to mycobacterial antigens in helminth-exposed individuals before and after bacilli Calmette-Guerin (BCG) vaccination. Clinical Experimental Immunology.

[17] WHO (2009b). Treatment of tuberculosis: guidelines.

[18] Sharma K, George N, Kadhiravan T, Saha K, Hemant KM, Hanif M (2009). Prevalence of extensively drug-resistant tuberculosis among patients with multidrug resistant tuberculosis: a retrospective hospital-based study. Indian Journal Medicine Resistance.

[19] WHO (2006a). Global Task Force Report on XDR-TB.

[20] WHO (2006b). Building on and enhancing DOTS to meet the TB-related Millennium Development Goals, The stop TB Strategy.

[21] WHO (2009a). Pathways to better diagnostics for tuberculosis: a blueprint for the development of TB diagnostics by the new diagnostics working group of the Stop TB Partnership.

[22] Shea YR, Davis JL, Huang L, Kovacs JA, Masur M, Mulindwa F (2009). High Sensitivity and Specificity of Acid-Fast Microscopy for Diagnosis of Pulmonary Tuberculosis in an African Population with a High Prevalence of Human Immunodeficiency Virus. Journal of Clinical Microbiology.

[23] WHO (2009e). AIDS epidemic update: November 2009.

[24] Cheesbrough (2009). District Laboratory Practice in Tropical Countries, Part 1 (Pt.1).

[25] Clerici M, Shearer GM (1994). The Thl/Th2 hypothesis of HIV infection. Immunol. Today.

[26] Leung GM, Rainer TH, Lau FL, Wong IO, Tong A, Wong TW, Kong JH, Hedley AJ, Lam TH (2004). Ann Intern Med.

